# Prevalence of the polymorphic H-f icolin (FCN3) genes and mannose-binding lectin-associated serine protease-2 (MASP2) in indigenous populations from the Russian Arctic regions

**DOI:** 10.18699/VJ21.098

**Published:** 2021-12

**Authors:** M.V. Smolnikova, S.Yu. Tereshchenko

**Affiliations:** Научно-исследовательский институт медицинских проблем Севера – обособленное подразделение Федерального исследовательского центра «Красноярский научный центр Сибирского отделения Российской академии наук», Красноярск, Россия; Научно-исследовательский институт медицинских проблем Севера – обособленное подразделение Федерального исследовательского центра «Красноярский научный центр Сибирского отделения Российской академии наук», Красноярск, Россия

**Keywords:** FCN3, MASP2, gene polymorphism, newborns, Russia, Arctic populations, FCN3, MASP2, полиморфизм генов, новорожденные, Россия, арктические популяции

## Abstract

Lectins, being the main proteins of the lectin pathway activating the complement system, are encoded by polymorphic genes, wherein point mutations cause the protein conformation and expression to change, which turns out to have an effect on the functionality and ability to respond to the pathogen. In the current study, largescale data on the population genotype distribution of the genes for H-ficolin FCN3 rs28357092 and mannose-binding lectin-associated serine protease MASP2 rs72550870 among the indigenous peoples of the Russian Arctic regions (Nenets, Dolgans and Nganasans, a mixed population and Russians: a total sample was about 1000 newborns) have been obtained for the first time. Genotyping was carried out using RT-PCR. The frequency of the homozygous variant del/del FCN3 rs28357092 associated with the total absence of the most powerful activator of the lectin complement pathway, N-ficolin, was revealed; 0 % in the Nenets, 0.8 % in the Dolgans and Nganasans, and 3.5 % among the Russians ( p < 0.01). Analysis of the prevalence of the MASP2 genotypes has shown the predominance of the homozygous variant AA in all studied populations, which agrees with the available world data. The heterozygous genotype AG rs72550870 associated with a reduced level of protease was found to occur rarely in the Nenets, Dolgans and Nganasans compared to newborns of Caucasoid origin from Krasnoyarsk: 0.5 % versus 3.3 %, respectively. Moreover, among 323 examined Nenets, one AG carrier was identified, whereas in Russians, 16 out of 242 examined newborns were found to be AG carriers ( p < 0.001). A homozygous variant (GG) in total absence of protease with impaired binding of both MBL and ficolins was not detected in any of the 980 examined newborns. An additional analysis of infectious morbidity in Arctic populations allows one to find phenotypic characteristics related to a high functional activity of the lectin pathway of complement activation as an most important factor for the first-line of anti-infectious defense, including such new viral diseases as COVID-19.

## Abstract (ru)

Лектины – основные протеины лектинового пути активации системы комплемента, кодируют- ся полиморфными генами, точечные мутации в которых приводят к изменению конформации и экспрессии белка, что в свою очередь отражается на функциональности и способности отвечать на патоген. В настоящем исследовании впервые получены масштабные данные о популяционном распределении частот аллелей ге- нов Н-фиколина FCN3 rs28357092 и маннозосвязывающей лектин-ассоциированной сериновой протеазы-2 MASP2 rs72550870 среди коренных народностей российских Арктических территорий (ненцы, долганы, нга- насаны, смешанная популяция и русские: общая выборка составила около 1000 новорожденных). Генотипи- рование осуществлено с использованием ПЦР-РВ. Нами выявлена частота гомозиготного варианта del/del FCN3 rs28357092, ассоциированного с полным отсутствием наиболее мощного активатора лектинового пути комплемента Н-фиколина: у ненцев 0 %, у долган-нганасан 0.8 %, в то время как среди европеоидов 3.5 % ( р <0.01). Анализ распространенности генотипов MASP2 показал преобладание гомозиготного варианта AA во всех исследованных популяциях, что согласуется с доступными мировыми данными. Гетерозиготный гено- тип AG rs72550870, ассоциированный со сниженным уровнем протеазы, встречается в единичных случаях у ненцев, долган и нганасан по сравнению с новорожденными европеоидного происхождения г. Красноярска: 0.5 и 3.3 % соответственно. Причем у ненцев был выявлен один носитель AG из 323 обследованных, тогда как у европеоидов – 16 из 242 обследованных новорожденных ( р < 0.001). Гомозиготный вариант GG, которому сопутствует полное отсутствие протеазы с нарушением связывания MBL и фиколинов, не обнаружен ни у од- ного из 980 обследованных новорожденных. Дополнительный анализ инфекционной заболеваемости в арктических популяциях позволит выявить фенотипические характеристики, сопряженные с высокой функцио- нальной активностью лектинового пути активации комплемента в роли важнейшего фактора первой линии противоинфекционной защиты, в том числе в отношении новых вирусных заболеваний, таких как COVID-19.

## Introduction

The innate immune system provides an immediate, non-specific first line of defense through humoral, cellular and mechanical processes, playing a vital role in protection against pathogenic effects (Dunkelberger, Song, 2010). In the world literature, considerable attention has recently been paid to the study of birth defects of the complement system (CS) in the pathogenesis of various infectious, autoimmune and cardio-metabolic diseases. Thus, in the document from European Society for Immunodeficiencies (ESID) 2020, specifically devoted to summarizing the current state of the problem of various complement component deficiencies, such birth defects were established to account for at least 5 % of the total number of primary immunodeficiencies, with their prevalence and pathogenesis being unexplored (Brodszki et al., 2020).

Plasma proteins of CS interact with each other in three known ways, i. e. lectin (the most phylogenetically ancient), alternative, and classical. All three complement pathways are initiated by many independent stimuli, and subsequently proteolytic cascades are reduced to the main component C3 activation, which leads to the assembly of the membrane-attacking complex (Blom et al., 2004). The lectin pathway (LP) can be activated in the absence of immune complexes and initiated by the binding of molecules of the pattern recognition receptor superfamily (lectins), such as mannose-binding lectin (MBL), colleсtin 11 (CL-K1), or ficolins, to carbohydrates or acetylated residues found on the pathogen surface or host apoptotic/cancer cells (Ali et al., 2012). Circulating MBL, CL-K1, and ficolins form complexes with specific serine proteases (mannose-binding lectin-associated serine protease, MASP).

In addition to complement activation, lectins reduce the risk of infection by stimulating the secretion of interferongamma (IFN-γ), IL-17, IL-6, tumor necrosis factor-alpha (TNF-α) by macrophages (Ren et al., 2014). Three types of ficolins have been described for humans: M-ficolin encoded by the FCN1 gene, L-ficolin (FCN2), and H-ficolin (FCN3). M-ficolin is expressed in lungs, monocytes and spleen, L-ficolin is produced in liver and circulates in blood, H-ficolin is expressed in liver and lungs. H-ficolin has been shown to be produced to the greatest extent in lungs, and its complement-activating ability exceeds that of MBL Ficolin-3 is the most abundant recognition molecule of the lectin pathway, and since it is highly expressed in liver and lung tissues, this indicates its importance both for activating the lectin pathway and for protecting the host lung (Akaiwa et al., 1999; Hummelshoj et al., 2008). In addition, the first evidence of antimicrobial activity of ficolin-3 against the intestinal commensal and opportunistic intestinal bacteria Hafnia alvei has recently been obtained (Michalski et al., 2015). It is noteworthy that ficolin-3 is resistant to collagenases (whereas other ficolins and collagens are not), and this may affect its antimicrobial activity, in particular in gastrointestinal tract (Hummelshoj et al., 2008).

Various polymorphic variants in the promoter and structural regions of ficolin genes were given. The FCN3 gene is located on chromosome 1p36.11 and is highly conserved in humans. Five point mutations responsible for amino acid substitutions have been described, with allele frequencies being below 5 %: p.Leu12Val, p.Leu117fs (known as +1637delC), p.Thr125Ala, p.Glu166Asp and p.Val287Ala (Hummelshoj et al., 2008). The high conservatism of the gene reveals that ficolin-3 may play a crucial role in the immune response. As a matter of fact, there have been very few cases of ficolin-3 deficiency (Thiel, 2007).

H-ficolin (ficolin-3) is the most potent of the known lectin complement pathway activators, and its serum concentrations are significantly higher than those of L-ficolin and MBL (Sallenbach et al., 2011). The rs28357092 (+1637delC) mutation in exon 5 of the FCN3 gene is a frame-shift mutation leading to truncation of the C-terminal end of the ficolin-3 protein; it accounts for a decrease in plasma levels of H- ficolin by the gene–effect scheme, i. e. homozygotes with such deletion demonstrate a total absence of H-ficolin plasma levels, and heterozygotes do moderate protein levels (Michalski et al., 2011). Homozygosity for +1637delC happens very rarely (1–2 %): only 6 cases have been described in the literature available (all of them suffered from severe infections in early childhood). Data on the population frequency of heterozygous carriage are also scarce, i. e. 15 heterozygotes out of 483 examined individuals were identified in the Icelandic cohort of healthy donors (the frequency was 1.5 %) (Bjarnadottir et al., 2016).

In addition to MBL and ficolins, one of the key participants in the lectin pathway of complement activation is the family of mannose-binding lectin-associated serine proteases; three proteases (MASP-1, MASP-2, MASP-3) and two related nonenzymatic proteins, MAp19 (sMAP) and MAp44 (MAP-1), were identified in the MASP family (Ricklin et al., 2010). MASP-1 and MASP-2 are of crucial importance in the lectin pathway activation. MASP-1 have been shown to be automatically activated and leads to the MASP-2 activation (Degn et al., 2012). MASP-2 can also become automatically activated, however, it is MASP-1 that is the main activator of MASP-2 under physiological conditions (Héja et al., 2012). MASP-2 is a protease that cleaves complement factors C2 and C4, leading to the complement cascade activation with the formation of inflammatory mediators (C3a and C5a), membrane attack complex (MAC) assembly and opsonization. On the other hand, MASP-3 seems to reduce the activity of the lectin pathway due to competition for MASP binding sites on the recognition molecules (Degn et al., 2010). Moreover, MASP-3 predominantly forms a complex with ficolin-3 and is believed to have an inhibitory effect on complement activation mediated by ficolin-3 (Skjoedt et al., 2010). Levels of the three MASPs have been demonstrated to be predictors of infection and prolonged dependence on life support in critically ill children (Ingels et al., 2014). The most studied among the specific enzymes capable of activating both MBL and ficolins is the type 2 prosthesis, i. e. MASP-2. Serum MASP-2 levels ranged from 125 to 1150 ng/ml, with an average of 416 ng/ml (Sallenbach et al., 2011). An analysis of plasma MASP-2 levels in people of various ethnic groups revealed that the lowest one was found in Africans, then the Hong Kong Chinese, Indians and Caucasian Danes (Thiel et al., 2007)

The polymorphic gene MASP2 is located on the 1p36.23- 31chromosome, has 12 exons, and encodes MASP-2 and MAp19 proteins. The most significant MASP2 mutation is rs72550870 (p.D120G), which leads to the substitution of aspartic acid for glycine, thereby the protein loses its ability to activate the complement due to inability to form lectin complexes, in particular with MBL and ficolins. Congenital MASP-2 deficiency is caused by the rs72550870 mutation in the homozygous state (GG), characterized by a total absence of serum protease activity (Thiel et al., 2009). A total of thirteen cases of homozygous GG rs72550870 have been described in the literature since the first case was detected in 2003 (Stengaard-Pedersen et al., 2003). Clinical manifestations of decreased activity/inactivity of MASP-2 can range from full health to severe infections and predisposition to cancer (Bjarnadottir et al., 2016). Since three healthy adults with MASP-2 deficiency, homozygous GG in MASP2, were reported (Garcia-Laorden et al., 2008), clinical penetrance of this deficiency has become doubtful. Thus, the association of MASP-2 deficiency (GG rs72550870) with clinical manifestations is currently uncertain. Unidentified molecules and functions are likely to be involved in the LP, which could explain why MASP-2 deficiency is relatively common in apparently healthy people (Bjarnadottir et al., 2016). The lectin pathway of the complement activation has been suggested to be unnecessary or also excessive (for example, in severe COVID) for an immune response in healthiest individuals to be formed, and its deficiency is clinically significant only in certain situations, for example, in premature infants (Matricardi et al., 2020).

There are some pronounced population differences in the genotype frequency distribution for polymorphic genes of lectin pathway proteins of the CS. The results of our earlier studies (Tereshchenko, Smolnikova, 2020) have demonstrated that the frequency of the high-producing haplotype (HYPA) of the MBL2 gene is 35.4 % in Russian newborns of Eastern Siberia, corresponding to that of European populations (Holland – 27 %, Denmark – 30 %, Czech Republic – 33 %), as well as Brazilian Caucasians (28–34 %). However, the HYPA haplotype frequency in newborns of the Taimyr Dolgan-Nenets region of the Krasnoyarsk Territory was statistically significantly higher than in the Russians and was 64 % for the Nenets and 56 % for the Dolgan-Nganasans, being close to the distribution frequency identified for the Eskimos (81 %) and the North American Indians (64 %). In the aboriginal populations of both the Nenets and Dolgans/ Nganasans of the Taimyr Dolgan-Nenets region of the Krasnoyarsk Territory, our research group found a decrease in the prevalence of the FCN2 rs7851696 genotype, associated with the low binding capacity of L-ficolin to carbohydrates, as compared with Caucasians of Eastern Siberia. The study results (Smolnikova et al., 2017) showed that the Nenets population has a number of important features compared to the Dolgans and Nganasans, i. e. a much lower prevalence of the T allele for the rs17549193 polymorphism and a higher prevalence of the T allele for the FCN2 rs7851696 polymorphism. We suppose that this genotype can be a genetic marker of the high functional ability of L-ficolin for the Nenets population. In other words, a high prevalence of genotypes associated with high L-ficolin activity in the Arctic populations of the Nenets and Dolgans/Nganasans, compared to the Caucasians of Eastern Siberia has been shown

As mentioned above, data on the population frequency of the rs28357092 polymorphic variants of the FCN3 gene are scarce, with much more works on the population frequencies for the rs72550870 polymorphisms of the MASP2 given. The frequency of the rare G allele in the Danish cohort was 3.9 %; the same frequency was found in the Icelandic sample of adult donors (Bjarnadottir et al., 2016). Interestingly, the G allele was not detected at all in the populations of Hong Kong Chinese, African Zambians, and Brazilian Native Americans (Fumagalli et al., 2017).

The results of the above studies underlie the hypothesis that human evolution has moved on to the accumulation of genotypes with low lectin pathway activity of complement activation due to the prevalence of some intracellular infections, such as tuberculosis and leprosy, where low MBL and L-ficolin activity can cause a protective effect (Verdu et al., 2006; Dunkelberger, Song, 2010). It was suggested that the isolated Arctic populations of the Taimyr Dolgan-Nenets region of the Krasnoyarsk Territory fought these infections historically later and, as a result, retained the high lectin pathway activity of complement activation formed at the early stages of human evolution.

According to the analysis of the literature data available, to date, the population frequencies of mutations associated with congenital deficiency of H-ficolin (rs28357092) and MASP-2 (rs72550870) in Russian populations and populations of indigenous peoples of the Russian Arctic regions have not been studied. The relevance of obtaining such data for the Russian Arctic populations is increasing, given the accumulating evidence that the lectin pathway of complement activation plays an important role in relation to viral infections. For example, MBL is assumed to be essential in relation to respiratory viral infections, including new coronavirus ones, i. e. SARS and COVID-19 (Matricardi et al., 2020). The role of congenital deficiencies of LP proteins, including H-ficolin and MASP-2, has not been studied in such clinical situations at all. Given that infections are major contributors to infant mortality and lectins are critical for anti-infectious defense, lectin deficiency is likely to contribute to early childhood mortality

The aim of the work was to identify ethnic differences in the allelic distribution of the lectin pathway component genes of complement activation among indigenous newborns of the Taimyr Dolgan-Nenets region of the Krasnoyarsk Territory (Nenets, Dolgans and Nganasans) compared to the Caucasians of Krasnoyarsk.

## Materials and methods

To study single nucleotide polymorphisms rs28357092 FCN3 and rs72550870 MASP2, 980 samples of dried blood stains from newborns from the Taimyr Dolgan-Nenets region of the Krasnoyarsk Territory and the city of Krasnoyarsk, obtained earlier in the Krasnoyarsk Regional Medical Genetic Center, were used.

The newborns were divided into four groups for the ethnic specificity of gene polymorphisms of the lectin pathway of the complement system to be studied: (1) 323 individuals from villages with a predominantly Nenets population (the Nenets make up 85 % of the population); (2) 138 from villages with predominantly Dolgan and Nganasan populations (the Dolgans and Nganasans make up 91 % of the population); (3) 217 from villages with varying combinations of indigenous and mixed populations; (4) 302 newborns of European origin from the city of Krasnoyarsk (the Russians).

Written informed consent of the subjects under the Helsinki Declaration of the World Association “Ethical Principles for Conducting Scientific Medical Research with Human Participation” as revised in 2000 and “The Rules of Clinical Practice in the Russian Federation”, adopted by Order of the Russian Ministry of Healthcare No. 266 dated June 19, 2003 was obtained for the investigation to be carried out. The study was approved by the Ethics Committee of Scientific Research Institute of Medical Problems of the North No. 9 dated September 9, 2014.

To isolate DNA from newborn blood stains, a DIAtom DNAPrep reagent kit (Isogen, Russia) was applied. Singlenucleotide polymorphism genotyping of the lectin pathway component genes of complement activation (FCN3, MASP2) was carried out under the manufacturer’s protocol, using the real-time polymerase chain reaction (RT-PCR) method with specific oligonucleotide primers and fluorescently labeled probes (TaqMan) (DNA-synthesis, Russia). Nucleotide sequences of allele-specific probes for genotyping polymorphisms are as follows: for rs28357092 FCN3 F – CCT CGGTGTCCATGTCAC, R – CCACCTTGAGCGGCTGG (fluorophore/allele – VIC/del, FAM/G); for rs72550870 MASP2 F – GCAAGGACACTTTCTACTCGC, R – TCA CCCTCGGCTGCATAG (fluorophore/allele – VIC/G, FAM/A).

The correspondence of genotype frequencies to the Hardy–Weinberg equilibrium was verified using χ2. Genotype frequency comparisons were performed using the twosided Fisher’s exact test. Statistically significant differences were accepted at p < 0.05 after Bonferroni correction for multiple tests.

## Results and discussion

The advantage of our approach to population assessment for the prevalence of immunodeficient genotypes of lectin pathway’s mediators of complement activation was to study newborn populations, where unfavorable genetic variations had not been excluded, which is possible at an older age as a result of the clinical realization of genetic predisposition.

The genotype frequencies and the variant allele of the H-ficolin gene FCN3 rs28357092 are given in Table 1. The prevalence analysis of the FCN3 genotypes revealed the predominance of the homozygous GG variant in all populations studied in the work, which is consistent with the world data available.

**Table 1.**
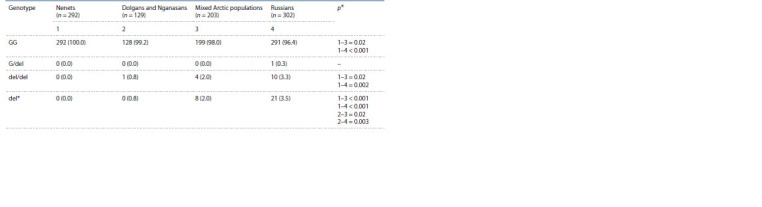
FCN3 rs28357092 genotype frequencies among newborns from different ethnic populations of the Taymyr Dolgan-Nenets region of the Krasnoyarsk Territory and the city of Krasnoyarsk, n (%) * Only p-values ≤ 0.05 are given.

The variant deletion allele (del) FCN3 rs28357092 in a heterozygous state was not found in any newborns of the three indigenous populations of the Taimyr Dolgano- Nenets region, except for one Russian individual from the city of Krasnoyarsk. Although the literature describes underrepresentation of the homozygous genotype for this deletion, it was found in our cohort of the studied samples in 10 Russian newborns (3.3 %), in 4 newborns from a mixed population (2.0 %) and in one of the Dolgan and Nganasan group (0.8 %). In the Nenets, neither homozygotes nor heterozygotes by the mutant deletion FCN3 rs28357092 were identified. Thus, in the total sample of 926 newborns, del/del homozygotes were detected in 15 individuals, being 1.6 %. According to the Internet source http://www.ensembl.org, the variant allele frequency in world populations is 1–3 %, with it being zero in Asian populations. As mentioned above, the rs28357092 (+1637delC) mutation in the FCN3 gene leads to a plasma level decrease of H-ficolin, i. e. the deletion homozygotes, being rare, have a complete absence of plasma H-ficolin, and heterozygotes have medium protein levels (Michalski et al., 2011; Bjarnadottir et al., 2016). It is likely that in other studies, homozygotes were not detected with adult populations being examined, demonstrating again the advantage of our approach for identifying the true genotype frequencies with a cohort of newborns, where the deficiency variants have not been eliminated due to infections and decease

The genotype frequencies and variant allele of the serine protease gene MASP2 rs72550870 are presented in Table 2. Analysis of the MASP2 genotype prevalence has shown the predominance of the homozygous AA variant among all populations studied in the work, which is consistent with the data available. The heterozygous genotype AG rs72550870 was found in some isolated incidents in the Nenets and Dolgan- Nganasans as compared with the Caucasian newborns in the city of Krasnoyarsk. The frequency of the AG genotype in the Russians (6.6 %) is statistically significantly higher than in the Arctic populations (the Nenets being 0.3 %, p < 0.001; the Dolgan-Nganasans being 1.4 %, p = 0.02; the mixed populations being 1.8 %, p = 0.01). Thus, the heterozygous AG variant occurred in 16 out of 242 Russian newborns, whereas in 323 Nenets it occurred in only one individual. None of the population groups were found to have homozygotes for the minor G allele associated with the absence of serum protease activity.

**Table 2.**
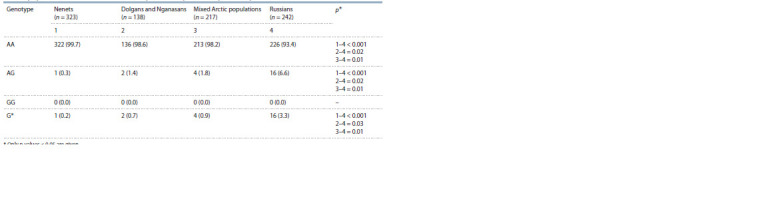
MASP2 rs72550870 genotype frequencies among newborns from different ethnic populations of the Taymyr Dolgan-Nenets region of the Krasnoyarsk Territory and the city of Krasnoyarsk, n (%) * Only p-values ≤ 0.05 are given.

The allelic variant G of the MASP2 rs72550870 has zero or extremely low frequencies in the world populations. According to the Internet source http://www.ensembl.org, the frequency in Caucasoid populations is 4.0 %, in the general American population – 2.0 %, among Asian and African populations – zero. In the course of the study, we obtained data on the prevalence of the mutant allele G rs72550870 in the Russian Arctic populations: 0.5 % among newborns in the Taimyr Dolgan-Nenets region of the Krasnoyarsk Territory (n = 678) and 3.3 % among Russians in Krasnoyarsk (n = 242).

Data on nine MASP2 gene mutations based on the two most informative studies carried out by the S. Triel group in 2007 and 2009 were obtained, that is, the prevalence of mutant allelic variants for almost all polymorphisms was too low. There was a change in the MASP-2 protein structure

that resulted from the rs72550870 mutation, which led to impaired binding into the MBL complex, resulting in the inability to activate the complement system. In addition, the authors note that it is the Caucasian population that has the variant G allele (rs72550870) as the main reason for the lower MASP-2 levels. Population analysis reported the absence of the homozygous genotype GG rs72550870 among the adult Chinese, Africans, Caucasians, Greenland Intuits, and Brazilians (Thiel et al., 2007, 2009). The heterozygous variant prevailed in the Caucasians from Denmark (3.9 %) and Inuits of western Greenland (where the European admixture is high, as reported by the authors) (3.7 %), however, that was not found to occur in other studied populations ( p < 0.0001).

Moreover, the authors (Thiel et al., 2009) provided the frequencies of a rare allelic variant obtained by other researchers in different populations among healthy individuals and patients with various diseases. Thus, 14 heterozygotes were found among 112 patients with cystic fibrosis (the frequency was 6.3 %) and five heterozygotes were found among 200 healthy people (the frequency – 1.3 %) in the Swedish population. In a study of psoriasis patients and their families, 894 individuals were tested for MASP2 rs72550870 and a total of 62 heterozygotes and one homozygote were found, resulting in a gene frequency of 3.6 % (the allele was not associated with psoriasis). Homozygosity was recorded in one person in a group of 293 Polish children with respiratory infections and in one child with cystic fibrosis. Two homozygotes were found among 2,008 individuals (including 967 pneumonia patients, 130 SLE patients, 43 children with recurrent respiratory infections, and 868 healthy people) in a recent study of the Spanish population, no association of the desease with the variant allele being found. The absence of the allelic variant G of the MASP2 rs72550870 in China was documented by a report examining the influence of both MBL2 and MASP2 genotypes on susceptibility to severe acute respiratory syndrome (SARS). No G allele was found in all 1,757 Asians tested. Thus, MBL-deficiency, as well as deficiencies of other complement components (including MASP2) should not be concluded to cause the disease or susceptibility to infections, but rather are clinical modifiers impairing other elements of the activation cascade.

Studying the role of congenital defects of the complement system in the pathogenesis of various diseases is a hot issue, as the congenital complement component deficiencies account for at least 5 % of the total number of primary immunodeficiencies, whereas their prevalence and pathogenesis remain unstudied. Large-scale data on the population genotype distribution of genes for H-ficolin FCN3 rs28357092 and mannose-binding lectin-associated serine protease MASP2 rs72550870 among the indigenous peoples of the Russian Arctic regions (the total sample of the newborns studied was 980) were first obtained in the work. As mentioned above, currently, the population frequencies of mutations associated with congenital deficiency of H-ficolin and MASP-2 in Russian populations as a whole, and in populations of indigenous peoples of the Russian Arctic regions in particular, have not been previously studied. Moreover, the previously identified features of the genetic regulation of lectin pathway proteins of complement activation in newborns of the Taimyr Dolgan- Nenets region of the Krasnoyarsk Territory have shown the indigenous populations of the Arctic to be genetically characterized by greater activity of at least two different lectin pathway components of complement activation, i. e. MBL and L-ficolin, indicating a high tone of the lectin pathway of complement activation in general (Smolnikova et al., 2017; Tereshchenko, Smolnikova, 2020).

Currently, there are two competing hypotheses accounting for the high genotype population diversity of the lectin compliment pathway (Eisen, Osthoff, 2014). The first of them proves to be a protective role of low-producing genotypes against some intracellular pathogens, i. e. tuberculosis and leprosy, visceral leishmaniasis, atypical pneumonia. A high level of lectin-mediated phagocytosis may predispose to better penetration of intracellular pathogens into the cytoplasm of host cells, screening the pathogens from factors of adaptive immunity and, consequently, a greater risk of active infection process. Thus, the actual data available suggest a “double pathophysiological role” of the lectin pathway of complement activation, i. e. protective against extracellular pathogens, especially in young children, and provocative against some intracellular pathogens and atherosclerosis. The population genetic consequences of such a “double role” may be the root cause of the ethnic diversity for the corresponding genotypes, being the main point for the first hypothesis mentioned above, based on the assumption for the selection benefit of the components deficiency of the lectin pathway of complement activation for some populations (Seyfarth et al., 2005; Eisen, Osthoff, 2014). The second hypothesis denies the existence of any selection pressure on the genotypes of the lectin complement pathway, accounting for genetic diversity specifically by migration processes and gene drift. However, the authors of the studies stipulate that “It is possible that stochastic evolutionary factors erased much of the ancient imprint left by natural selection and more powerful tests in greater population samples would be necessary to confirm the data” (Verdu et al., 2006; Boldt et al., 2010).

## Conclusion

Therefore, lower prevalence of the genetic markers of the H-ficolin and MASP-2 deficiencies in the indigenous populations of the Arctic regions of the Krasnoyarsk Territory as compared with the Caucasians of Krasnoyarsk city, associated with a genetic predisposition to a high functional activity of L-ficolin compared to Caucasian population, has been expected to be revealed in the given work.

The study of the ethnically associated non-specific antiinfectious protection among the indigenous population of the Taimyr Dolgan-Nenets region of the Krasnoyarsk Territory can be used to formulate plans for practical health authorities towards the infection prevention and in order to efficiently attract labor resources for working in conditions with a high infectious load. An additional analysis of infectious morbidity among the Arctic populations will allow one to reveal phenotypic characteristics associated with a high functional activity of the lectin pathway of complement activation as the most important factor for the first line of anti-infectious defense, including such new viral diseases as COVID-19. Such clinical and genetic comparisons are extremely important for elucidating the physiological role of MBL, ficolins, and MASP-2, and we identified genetic features of the ethnically isolated indigenous Arctic populations of the Krasnoyarsk Territory as a unique material to be studied.

## Conflict of interest

The authors declare no conflict of interest.
